# Innovative Technologies: Cellular Jigsaw Puzzles

**DOI:** 10.1289/ehp.112-1247666

**Published:** 2004-11

**Authors:** Richard Dahl

Although scientists have great understanding of individual molecules, the limitations of modern technology have restricted the study of molecular groupings, or “machines,” within cells. Now, however, scientists with the Structural and Computational Biology Programme at the European Molecular Biology Laboratory (EMBL) in Heidelberg, Germany, have developed a method to predict and gain understanding of how molecules assemble into machines—an advancement with significant potential application to toxicogenomics.

Group leader Rob Russell says advances in the study of functional genomics in recent years have provided the basis for knowing what components make up these molecular machines, even though little is known about what the structures look like. This new knowledge about the makeup of molecular complexes, combined with electron microscopy technology and computer methods developed by Russell and computational biologist Patrick Aloy, provided the framework for the project.

Russell and Aloy studied yeast proteins, identifying the components of hundreds of molecular machines in these cells. Using the “tandem affinity purification” method developed at EMBL Heidelberg, they attached molecular tags to selected proteins and “went fishing” for other proteins in the yeast that would interact with the bait. These interactive complexes form the basis of protein networks.

They liken the process, from that point on, to that of assembling a jigsaw puzzle, where the pieces are individual components of particular machines in yeast cells. They first divided the components into groups containing structural similarities, then proceeded to look for recurring patterns of molecular interactions. For example, if two similar molecules in one machine were also found in another, they were considered likely to fit together in the same way.

The scientists looked for those kinds of relationships and built upward, using knowledge about how various protein molecules fit together in one machine to predict the structure of other machines. In some cases, they were able to draw three-dimensional images of machines on their computer screens. Aloy cautions, however, that these images are predictions—not depictions—of structure.

The potential application of the research at EMBL Heidelberg, which was conducted in conjunction with the private German biopharmaceutical company Cellzome, may be broad. “If you know something about structure, you know a lot about how something works,” Russell says. “If you’re confident that the structure is right, you could conceivably design chemicals to target particular types of machines.”

Andrej Sali, a professor at the University of California, San Francisco, departments of Biopharmaceutical Sciences and Pharmaceutical Chemistry, says that scientists working on structural genomics have become keenly interested in how protein assemblies function. “The general point is that structures of assemblies are informative about what the function of the assemblies is and how that function is performed mechanistically—how one might want to control that function, or modify it, and perhaps eventually how one could design new functions,” Sali says. “So, for these purposes, knowledge of structure is very helpful.”

Sali says Russell and Aloy’s report of their research, which appeared in the 26 March 2004 issue of *Science*, has been widely read because it presents a new way to envision molecular structures as systems that appear in three dimensions, and not just as individual proteins.

Russell believes that knowledge of molecular machines is useful in toxicogenomics because so much in this science relies on being able to understand the relationship between often highly disparate processes. “For instance,” he says, “how does liver hyperplasia arise when one is taking a drug acting on a particular kinase? This essentially boils down to understanding the relationship between pathways in the cell, and certainly a structural perspective on this can be a great boon.”

Russell says he and his colleagues have only begun to scratch the surface with their work on the functions of molecular machines. EMBL Heidelberg, which is funded by public research monies from 17 member states, recently received a grant from the Sixth Framework Programme of the European Community (which funds research, development, and demonstration activities) to carry the work to the next level. Russell says his laboratory will be working with approximately 20 other groups in Europe to embark on a variety of further experiments using tools including electron microscopy and X-ray crystallography. “The hope,” he says, “is to do this in more detail than what we were able to do in the original paper.”

Although there is obviously much work left to do to realize the potential of this new method, the possibilities appear wide open. “It’s an exciting area,” Russell says. “Our ultimate goal is to have a kind of dynamically updated view of the cell. . . . Ultimately, we want a complete picture of the cell.”

## Figures and Tables

**Figure:**
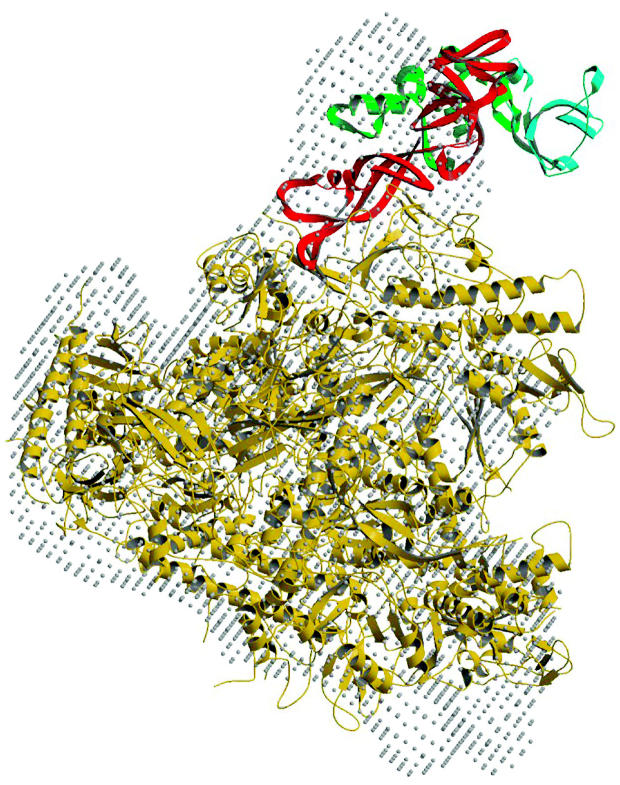
**Pieces of the puzzle.** Researchers at EMBL Heidelberg have devised a way to apply functional genomics to derive the structure of a molecular machine within the yeast cell. Such models may also suggest a potential mode of interaction between polymerase and transcription initiation factors.

